# Blended learning: Assessing nursing students’ perspectives

**DOI:** 10.4102/curationis.v47i1.2579

**Published:** 2024-07-05

**Authors:** Ilze Steenkamp, Jennifer Chipps

**Affiliations:** 1 Department of Nursing, Faculty of Community and Health Sciences, University of the Western Cape, Cape Town, South Africa

**Keywords:** blended learning, COVID-19, online, perceptions, nursing students

## Abstract

**Background:**

Blended learning combines face-to-face and online learning and has recently gained popularity, accelerated by the coronavirus disease 2019 (COVID-19) pandemic, often without active evaluation.

**Objectives:**

This study aimed to assess university nursing students’ perceptions of a blended learning approach during the COVID-19 pandemic.

**Method:**

The population was 150 third-year nursing students from a university in the Western Cape, South Africa, using all-inclusive sampling. A one-group, pre-and post-evaluation study was conducted using a self-administered questionnaire (Student Perceptions of Blended Learning scale). Differences were assessed using Chi-squared and Mann–Whitney U tests with a significance level of *p* < 0.05.

**Results:**

Before implementing blended learning, 128 students (85.3%) completed the questionnaire, while 95 (63.3%) did so after. Demographics and access showed no significant differences between the groups. Post-implementation showed a 10.1% increase in preference for blended learning (χ2 = 2.832, *p* = 0.092). Ease of use was rated significantly higher before implementation (3.07 ± 0.49), with no significant change post-implementation (2.99 ± 0.58). The blended learning process received lower ratings compared to content, with no significant differences before or after implementation for either (process: 2.55 ± 0.58 vs 2.54 ± 0.63; content: 2.75 ± 0.52 vs 2.79 ± 0.52).

**Conclusion:**

Nursing students had a positive perception of blended learning, though the online learning component posed challenges with time and module comprehension.

**Contribution:**

The findings can help higher education institutions evaluate existing online management systems and guide nurse educators in meeting students’ needs when developing module resources.

## Introduction and background

Blended learning is an educational approach that combines traditional classroom, face-to-face teaching with the use of digital resources and online learning (Al-Osaimi & Fawaz 2022; Hsu 2011; Lothridge, Fox & Fynan 2013; Sung, Kwon & Ryu 2008). Blended learning has become increasingly popular in education over recent years, fast-tracked by the coronavirus disease 2019 (COVID-19) pandemic (Al-Osaimi & Fawaz 2022; Divjak et al. 2022; Singh, Steele & Singh 2021; Su et al. 2023). The pandemic highlighted the importance of higher educational institutions to support blended learning by including high-quality online resources together with support for students, ensuring a positive and productive learning experience (Kanagaraj et al. 2022; Kumar et al. 2021; Mukasa et al. 2021).

Blended learning is a practical approach to education with reported high satisfaction levels making it relevant in the training of nurses (Howlett et al. 2011; Knox, Cullen & Dunne 2013; Kumar et al. 2021; Makhdoom et al. 2013; Wu, Tennyson & Hsia 2010). With blended learning, the online component allows students to be flexible to learn at their own pace and in their own time and have access to a variety of learning materials (Shang & Liu 2018; Singh et al. 2021).

Blended learning is known for promoting student engagement and active involvement (Lu 2021; Mukasa et al. 2021; Shang & Liu 2018; Swaminathan et al. 2021). The level of student participation in blended learning depends on a number of factors such as the quality of online content, educators’ presence and rapport, flexibility of the teaching schedule, home environments, access to technology and personal qualities such as self-discipline (Al-Osaimi & Fawaz 2022; Kanagaraj et al. 2022; Kumar et al. 2021; Langegård et al. 2021; Su et al. 2023).

To facilitate a blended learning environment, it is essential for nurse educators to establish an immersive learning environment that allows students to actively participate with the content and interact meaningfully with their peers (El-Zeftawy & Hassan 2016; Poon 2013; Kumar et al. 2021). When executed effectively, blended learning can transform the traditional classroom into a dynamic space where students are encouraged to collaborate and think critically about complex concepts, resulting in opportunities for personal growth and academic success (El-Zeftawy & Hassan 2016; Poon 2013; Kumar et al. 2021).

While blended learning offers numerous benefits, its success depends on students’ perceptions and acceptance of the approach. It is important to gain insight into how students perceive blended learning and to identify challenges, opportunities and appropriate educational strategies to maximise learning outcomes (Herbert et al. 2017; Kanagaraj et al. 2022; Min & Yu 2023; Muhtia, Suparno & Sumardi 2019; Wright 2017).

While most universities underwent a significant transition to blended learning amid the pandemic, there is a lack of evidence regarding the perspectives of nursing students on the blended learning approach and its influence on their learning experiences during the pandemic. This study aimed to evaluate the nursing students’ perceptions of blended learning processes, learning content and ease of use pre and post-implementation of a blended learning approach in a specific learning module.

## Blended learning approach

In response to the global pandemic, a blended education approach was implemented with third-year nursing students enrolled in a community health module, by including online teaching to prioritise the safety of students. The aim was to balance face-to-face and online learning interactions to minimise in-person contact and mitigate the risk of COVID-19 transmission. Blended learning was implemented from February to June 2021. To ensure a smooth transition, students underwent a 1-week blended learning orientation at the beginning of the semester (February 2021). The orientation covered the fundamentals of the blended learning approach and its application in the module, emphasising how this approach would support student learning under the circumstances at that time (Table 1).

**TABLE 1:** Blended learning orientation.

Orientation Part 1: Orientation to online learning (online session) Orientation to online learning (using an online meeting platform) University online learning management system Module outline and lessons on online learning management system Guidance on open-source applications (including online class meeting tools) Virtual visit from librarian illustrating remote access Practice using the online tools, including audio and microphone checks Dealing with online technical challenges (audio and video) and referral to campus IT support
Orientation Part 2: Preparation for pandemic (face-to-face session) On-campus session (adaptation of face-to-face instruction) tailored to the pandemic Orientation to using small group sessions and staggered contact times, along with the implementation of protective measures The on-campus session took place within a carefully managed setting, where precautions were implemented

## Research methods and design

### Setting

The study was conducted at a nursing school at a university in the Western Cape, South Africa. The school offers a 4-year undergraduate degree in nursing. Students were already exposed to a variety of educational approaches, for example, online learning management systems, clinical skills laboratory and traditional face-to-face teaching. But at the time of the study, there was no formal blended learning approach built into the nursing programme.

### Study design

A one-group pre- and post-evaluation study using pre- and post-surveys using a self-administered questionnaire was conducted to assess nursing students’ perceptions of using a blended learning approach during the pandemic.

### Study population and sampling strategy

The target population was 150 third-year nursing students enrolled in the undergraduate nursing programme for a selected third-year module. All-inclusive sampling was used, and all the third-year students were given the questionnaires to complete at both times of data collection. This group of students was specifically chosen for this study because of their unique position in their academic journey, having completed 2 years of traditional face-to-face classes, prior to the challenges brought about by the COVID-19 pandemic and then having to adapt to a blended learning approach with online lectures and face-to-face clinical training.

### Instrument

A self-administered questionnaire was used to collect pre- and post-group data with a validated and reliable scale on student perceptions of blended learning (Shantakumari & Sajith 2015). The scale has 24 statements covering three domains in blended learning; process, content and ease of use (Shantakumari & Sajith 2015). Each statement was rated on a scale from 0 to 4 ranging from strongly disagree (0) to strongly agree (4). The questionnaire was in English and was piloted with five nursing students, resulting in no changes to the scale and the inclusion of the pretest data. The scale has good internal consistency (α = 0.87) (Shantakumari & Sajith 2015), similarly to this study (α = 0.87), with the three domains also showing good internal consistency, process (α = 0.81), content (α = 0.73) and ease of use (α = 0.51).

### Data collection

The pre-survey data collection was done during the orientation week in February 2021 prior to implementation of the community health nursing module. The post-data were collected using the same questionnaire, post-completion of the module in June 2021, during the last clinical skills session. Completion of the questionnaires was voluntary, anonymous and took 5 min to complete. Written informed consent was obtained from respondents of the study.

### Data analysis

Data were captured and analysed in SPSS v28.0 (IBM Corporation, Armonk, New York, US). Students’ perceptions of the three domains were analysed using means and standard deviations as per scale instructions for each of the domains and for the individual items. When analysing the responses of individual items in the questionnaire, a mean score of 2 was used as a reference value to analyse perceptions. A score of 2 or more for an item was regarded as positive, while less than 2 was considered negative (Shantakumari & Sajith 2015). Statements that were negatively phrased were reversed. The overall scale had a maximum attainable score of 96, with higher scores meaning more positive perceptions. Perceptions were classified into three categories that reflect the extent of the positive perceptions related to blended learning: 28–53, i.e. relatively low level of positive perceptions, possibly indicating reservations or concerns; 54–80, i.e. moderate to high levels of positive perceptions, possibly finding value in blended learning and > 80, reflecting a high level of positive perceptions and possibly strong endorsement and satisfaction (Shantakumari & Sajith 2015). Independent samples Chi-squared tests were performed to determine differences between pre- and post-survey demographics. Independent samples Mann–Whitney *U* tests were performed to determine whether there were differences in the mean scores of the students’ perception of blended learning pre and post-implementation of the module. The significance level was set as *p* < 0.05.

### Ethical considerations

Ethics approval was obtained from the University of the Western Cape Humanities and Social Sciences Research Ethics Committee (HSSREC), reference number HS21/3/2, and permission to conduct the study was obtained from the university registrar and the director of the nursing school. Following the ethics approval, information about the study was distributed to third-year students. Written informed consent was obtained from those respondents who agreed to participate. Students were free to voluntarily participate or withdraw without penalty. Questionnaires were coded and anonymous. All data were securely stored.

## Results

### Demographics

The questionnaire was completed by 128 (85.3%) third-year nursing students prior to the implementation of the module and 95 (63.3%) respondents after the module was completed. There were no significant differences observed in demographics between the pre- and post-group respondents with most of the respondents being female (113, 88.3% pre-group; 84, 88.4% post-group), and the majority were 24 years and younger (105, 82.7% pre-group; 79, 84.0% post-group) (Table 2).

**TABLE 2:** Blended learning respondent demographics.

Demographic variables	Pre- vs Post-group		*χ* ^*2*^ †	*p*
	*n*	%		
**Age**				
< 24 years	105 vs 79	82.7 vs 84.0	0.072	0.788
**Gender**				
Male	15 vs 11	11.7 vs 11.6	0.001	0.974
Female	113 vs 84	88.3 vs 88.4		

*p* < 0.05.

† , *χ*^2^ Fisher’s exact tests.

### Technology access

There were no significant differences in technology access during the implementation with more than half of the respondents (70, 54.7% pre-group; 60, 62.2% post-group) reporting that they had a laptop for accessing study modules and most reporting that they owned a cell phone with Internet access (120, 95.2% pre-group; 93, 97.9% post-group) and Internet at home (108, 86.4% pre-group; 78, 83.9% post-group). Email appeared to be the preferred mode of communication (83, 66.4% pre-group; 68, 73.1% post-group) (Table 3).

**TABLE 3:** Blended learning technology access.

Technology access	Pre- vs Post-group		*χ* ^*2*^ †	*p*
	*n*	%		
Cell phone with Internet access	120 vs 93	95.2 vs 97.9	0.471	0.252
**Primary device to access module work**				
Laptop	70 vs 60	54.7 vs 62.2	0.445	0.436
Cell phone	55 vs 33	43.0 vs 34.7		
Desktop	2 vs 2	2.3 vs 2.1		
Internet access at the place of residence	108 vs 78	86.4 vs 83.9	0.272	0.602
**Preferred communication method from lecturer**				
Email	83 vs 68	66.4 vs 73.1	1.413	0.493
WhatsApp group	33 vs 21	26.4 vs 22.6		
Learning management system notices and messages	9 vs 4	7.2 vs 4.3		

*p* < 0.05.

† , χ^2^ Fisher’s exact tests.

### Overall perception of blended learning

Before the implementation of blended learning, over two-thirds of the respondents indicated that they would prefer the blended learning approach over traditional face-to-face learning (86, 68.8%). This remained so post-implementation of blended learning with a near significant increase of 10.1% (75, 78.9%; *χ*^*2*^ = 2.832, *p* = 0.092).

**FIGURE 1:**
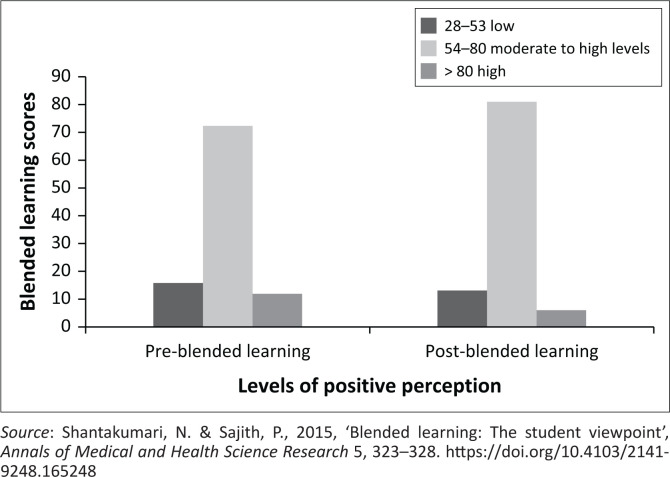
Categories of levels of perception pre- and post-blended learning.

There was no significant difference in the overall blended learning score pre and post the blended learning implementation (pre 64.96/95, ± 11.47 vs. post 64.60/95, *d* = –0.36 ± 11.59; *W* = –0.11, *p* = 0.014). Around three-quarters of the respondents’ score were in the 54–80 group, i.e., moderate to high levels of positive perceptions, increasing post-implementation (73, 72.3% pre-group; 68, 81.2% post-group), because of a decrease of 5.6% in the score post-implementation in the > 80 group (12, 11.9% pre-group; 5, 6.0% post-group), i.e., high level of positive perceptions (Figure 1).

### Students’ perception of blended learning domains

Perceptions of blended learning were measured in terms of the blended learning process (8 positive and 2 negative statements, Table 4), blended learning content (9 positive and 1 negative statements, Table 5) and blended learning ease of use (4 positive and 1 negative statement Table 6), with each statement rated out of a possible 4.

**TABLE 4:** Perceptions of the blended learning process.

Items	Pre-Mean ± s.d. *n* = 128	Post-Mean ± s.d. *n* = 95	*U*	*p*
I am in favour of incorporating blended learning to my module	3.11 ± 0.91	2.89 ± 1.01	−1.71	0.088
Blended learning encouraged me to learn	3.04 ± 0.87	2.76 ± 0.93	−2.36	0.018
Incorporating blended learning has deepened my interest in the subject matter of this module	2.80 ± 0.93	2.66 ± 0.88	−1.38	0.168
Blended learning is less stressful than traditional in class delivery	2.72 ± 1.27	2.71 ± 1.18	−0.27	0.785
I received adequate assistance in case of problems faced	2.72 ± 0.89	2.60 ± 0.98	−0.73	0.466
Blended learning in my module improved my interaction with the lecturer	2.62 ± 1.09	2.59 ± 1.05	−0.36	0.720
Blended learning in my module improved my interaction with my colleagues	2.61 ± 1.08	2.67 ± 1.05	0.39	0.697
Blended learning is more effective than traditional in class delivery	2.56 ± 1.17	2.58 ± 1.05	−0.06	0.950
Blended learning is a waste of time†	3.13 ± 1.05	2.43 ± 1.33	−3.93	< 0.001
Incorporation of blended learning made my module more time consuming†	1.59 ± 1.07	1.52 ± 1.09	−0.80	0.424
Overall mean of process domain	2.55* ± 0.58	2.54/4 ± 0.63	0.03	0.978

*Source:* Shantakumari, N. & Sajith, P., 2015, ‘Blended learning: The student viewpoint’, *Annals of Medical and Health Science Research* 5, 323–328. https://doi.org/10.4103/2141-9248.165248

Note: Emphasis added to highlight overall mean.

s.d., standard deviation; *U*, independent samples Mann-Whitney U test.

† , Negatively worded item in questionnaire.

* , average score in the process out of 4.

**TABLE 5:** Perceptions of the blended learning content.

Items	Pre-Mean *n* = 128	s.d	Post Mean *n* = 95	s.d	*U*	*p*
The online activities on the learning management system were related to the module objectives	3.27	0.73	3.14	0.69	−1.49	0.136
The online content was well illustrated	2.98	0.78	2.92	0.86	−0.41	0.683
The online content was easy to understand	2.95	0.89	2.95	0.88	−0.58	0.957
The blended learning content encouraged me to learn	2.93	0.89	2.87	0.85	−0.58	0.561
I learned a great deal from this module	2.88	0.79	2.98	0.80	0.94	0.348
The assignments given in the module helped me develop my writing skills	2.83	0.89	2.90	0.88	0.74	0.457
The activities of blended learning helped me to learn more and interact with my colleagues on the given topics	2.64	0.99	2.74	0.96	0.78	0.434
The entire module was difficult to follow†	2.41	1.16	2.05	1.19	−2.19	0.028
The online activities were of long duration†	1.48	0.96	1.29	0.92	−1.49	0.135
Overall mean of content domain	2.75/4	0.52	2.79/4	0.52	0.90	0.369

*Source*: Shantakumari, N. & Sajith, P., 2015, ‘Blended learning: The student viewpoint’, *Annals of Medical and Health Science Research* 5, 323–328. https://doi.org/10.4103/2141-9248.165248

Note: Emphasis added to highlight overall mean.

*U*, Independent samples Mann-Whitney *U* test.

† , Negatively worded item in questionnaire.

**TABLE 6:** Blended learning ease of use.

Items	Pre-Mean *n* = 128	s.d.	Post-Mean *n* = 95	s.d.	*U*	*p*
I found the learning management system easy to use	3.61	0.65	3.44	0.67	−2.16	0.030
I was able to learn to use the learning management system quickly	3.56	1.16	3.34	0.74	−2.17	0.030
I was able to access the online content without any problems	3.29	0.81	3.01	0.94	−2.19	0.029
My computer skills have improved as a result of this module	3.24	0.88	3.13	0.79	−1.39	0.166
I felt my knowledge regarding using the learning management system was limited compared to my peers†	2.34	1.36	1.93	1.34	−2.15	0.032
Overall mean of ease-of-use domain	3.07/4	0.49	2.99/4	0.58	−1.24	0.214

*Source:* Shantakumari, N. & Sajith, P., 2015, ‘Blended learning: The student viewpoint’, *Annals of Medical and Health Science Research* 5, 323–328. https://doi.org/10.4103/2141-9248.165248

Note: Emphasis added to highlight overall mean.

*U*, Independent Samples Mann-Whitney *U* test.

† , Negatively worded item in questionnaire

**FIGURE 2:**
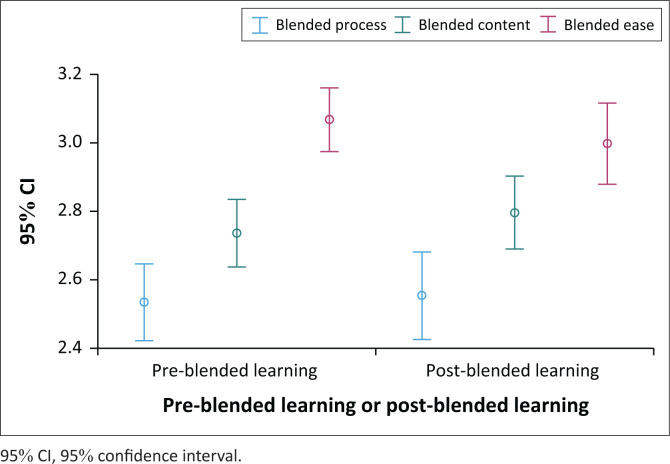
Blended learning process across pre-and post-implementation.

All domains had a score above 2, i.e., positive perceptions of the blended learning process (pre-group 2.55, ± 0.58; post-group 2.54, ± 0.63), content (pre-group 2.75, ± 0.52; post-group 2.79, ± 0.52) and ease of use (pre-group 3.07, ± 0.49; post-group 2.99, ± 0.58), with no significant differences in the overall rating score pre and post. However, the blended learning process domain was rated significantly lower on both occasions compared to score for content delivered via blended learning and ease of use, which was rated significantly higher in the pre-measurement (Figure 2).

### Blended learning process

Overall, the process domain was rated significantly lower than the other domains (Figure 2), though the overall score was above 2. The statement ‘I am in favour of incorporating blended learning to my module’ had the highest level of agreement (pre-group 3.11, ± 0.91; post-group 2.89, ± 1.01; *U* = –1.71, *p* = 0.088), though there was no significant difference post-implementation. The statement ‘Blended learning is more effective than traditional in class delivery’ had the lowest level of agreement (pre-group 2.56, ± 1.17; post-group 2.58, ± 1.33; *U* = –0.06, *p* = 0.950), with no significant difference post-implementation (Table 4).

In terms of the negative statements, ‘Blended learning is a waste of time’ had the highest agreement ratings from respondents, though significantly lower post-implementation (pre-group 3.13 ± 1.05; post-group 2.43 ± 1.33; *U* = –3.93, *p* < 0.001). ‘Incorporation of blended learning made my module more time-consuming’ had a low level of agreement (pre-group 1.59 ± 1.07 vs. post-group 1.52 ± 1.09; *U* = –0.80, *p* = 0.422), which did not differ significantly post-implementation (Table 4).

### Blended learning content

Overall, the blended learning content domain was rated significantly higher than the process domain (pre- and post-implementation) but lower than the ease-of-use domain, significantly so pre-implementation (Figure 2). In terms of the positive statements, ‘The online activities on the learning management system were related to the module objectives’ had the highest agreement rating both pre and post-implementation (pre-group 3.27, ± 0.73 vs post-group 3.14, ± 0.69; *U* = –1.49, *p* = 0.136). The statement, ‘The activities of blended learning helped me to learn more and interact with my colleagues on the given topics’, had the lowest agreement ratings pre and post-implementation, though the post ratings were higher though not significant. All ratings were above 2 indicating positive perceptions of the content taught using blended learning (Table 5).

In terms of the negative statements, ‘The entire module was difficult to follow’ had a high level of agreement (above 2) but significantly lower post-implementation (pre-group 2.41 ± 1.16; post-group 2.05 ± 1.19; *U* = –2.19, *p* = 0.028). The statement ‘The online activities were of long duration’ had low level of agreement (below 2) and decreased post-implementation though not significantly (pre-group 1.48, ± 0.96 vs. post-group 1.29, ± 0.92; *U* = –1.49, *p* = 0.135) (Table 5).

### Blended learning ease of use

Overall, the ease-of-use domain was rated higher than the other domains, significantly more so before the implementation (Figure 2) with all the positive statements rated over 3 (Table 6). The statement ‘I found the learning management system easy to use’ was rated the highest pre (3.61, ± 0.65) and post-implementation (3.44, ± 0.67; *U* = –2.16, *p* = 0.030), though significantly lower post-implementation. Except for two items, ‘I was able to learn to use the learning management system quickly’ (pre-group 3.56 ± 1.16; post-group 3.34 ± 0.74; *U* = –2.17, *p* = 0.030) and ‘I was able to access the online content without any problems’ (pre-group 3.29 ± 0.81; post-group 3.01 ± 0.94; *U* = –2.19, *p* = 0.029), which both decreased significantly post-implementation, there were no significant differences in pre- and post-survey ratings for these items (Table 6).

The negative statement, ‘I felt my knowledge regarding using the learning management system was limited compared to my peers’ was rated significantly lower post-implementation, with the agreement dropping significantly post-implementation (pre-group 2.34, ± 1.36 vs. post-group 1.93, ± 1.34; *U* = –2.15, *p* = 0.032).

## Discussion

The study investigated nursing students’ perceptions in using a blended learning approach during the pandemic implemented in a specific module. Blended learning was evaluated against three domains, the process of implementation, the content delivered via blended learning and the ease of use. In general, there was a positive perception towards the implementation and use of blended learning among respondents, which increased post-implementation with respondents still preferring blended learning after the module, with an increase (pre-group 86, 68.8%; post-group 75, 78.9%). This is consistent with other studies’ findings, which support the general favourable opinion of blended learning (Khan et al. 2021; Langegård et al. 2021; Lu 2021). The strong technology foundation is evident in the high number of respondents indicating access to cell phones and Internet connectivity with a preference for e-mail communication.

### Blended learning process

Though the blended learning process was rated significantly lower than the other domains, all the statements were positive about the process. There was some concern prior to the implementation that incorporating blended learning could potentially lead to time wastage (3.13, ± 1.05) though this dropped significantly post-implementation (2.43, ± 1.33). This was similar to concerns regarding the use of blended learning reported by other studies (Almahasees, Mohsen & Amin 2021; Lu 2021; Siraj & Maskari 2019; Wu et al. 2020), where it was hypothesised that perceived time wasting might be affected by factors such as the online structure of components, first-time user difficulties, lengthy resources such as videos (Siraj & Maskari 2019; Su et al. 2023; Swaminathan et al. 2021). Suggestions to address this perception and to improve comprehension of the content while also making the best use of the time spent by students include shorter videos, engaging activities (Armellini, Teixeira Antunes & Howe 2021; Becker et al. 2017; Siraj & Maskari 2019; Su et al. 2023; Swaminathan et al. 2021) and streamlining the content to ensure clear, engaging and goal-oriented educational resources (Almahasees et al. 2021; Su et al. 2023). In addition, asynchronous and flexible learning schedules allow students to engage with content at their convenience, reducing time waste and enhancing inclusion and accessibility in blended learning together with detailed timetables, targets and reminders (Almahasees et al. 2021; Armellini et al. 2021; Becker et al. 2017; Lu 2021; Su et al. 2023).

### Blended learning content

Overall, the content delivered via blended learning domain was rated positively with the design of the module and respondents’ comprehension of the resources influencing their perception of blended learning. The alignment of online exercises with module objectives received mostly positive feedback pre- and post-implementation (pre-group, 3.27 ± 0.73; post-group, 3.14 ± 0.69, ns). This was like findings in blended learning literature where participants expressed an interest in learning through online content, which allows them to access materials as needed, and the given content should be appealing and simple to grasp (Alexander & Boud 2018; Larson & Lockee 2019; Lu 2021; Swaminathan et al. 2021).

The respondents found the module difficult to follow (2.41, ± 1.16) pre-implementation, which reduced significantly post-implementation (2.05, ± 1.19), though this remained high with an agreement level over 2. This aligns with similar findings in another study (Langegård et al. 2021), which attributed this finding, to the nurse educators’ limited knowledge of technology, learning management systems and their ability to create comprehensive online content. A lack of experience might lead them to rely on methods traditionally used in face-to-face settings, such as presenting a slideshow lecture, without fully utilising the features of the learning management system or incorporating a more diverse range of online resources. Similar findings from other studies suggest that educators working in online environments face similar difficulties (Dailey-Hebert 2018; Johnson, Mejia & Cook 2015; Larson & Lockee 2019).

These findings suggest there is a need for clearer instructional design and communication strategies to improve content accessibility and understanding (Langegård et al. 2021; Lu 2021; Singh et al. 2021). This can be done by refining the module design to prioritise clarity and engagement. In addition, offering additional resources or support mechanisms can enhance participants’ understanding (Khan et al. 2021). Actively seeking and integrating student feedback on materials in the module is essential for ongoing improvement and alignment with students’ needs (Khan et al. 2021). In addition, nurse educators require consistent training opportunities and skill development if blended learning is to continue to be effective (Almahasees et al. 2021; Jowsey et al. 2020; Singh et al. 2021; Siraj & Maskari 2019).

### Blended learning ease of use

The ease of use was measured in terms of the use of the learning management system, and this had high levels of agreement of the ease of use of the learning management system, pre- and post-implementation. However, concerns were expressed regarding self-perceived knowledge limitations compared to peers (pre-group 2.34, ± 1.36; post-group 1.93, ± 1.34) pre the module, which reduced significantly post-implementation. This suggests a potential gap in students’ confidence and online competence prior to the implementation of blended learning, which can play an important role in effectively using an online learning management system (Nwamu & Ni 2023; Singh et al. 2021). This also highlights the importance of ongoing support and training initiatives (Rasheed, Kamsin & Abdullah 2020; Siraj & Maskari 2019) prior to implementing blended learning. Training and preparation to ensure students have necessary skills and knowledge to sidestep difficulties, as was done in this study, can enhance the overall experience of using blended learning (Divjak et al. 2022; Khan et al. 2021; Singh et al. 2021). Other recommendations for training should also include accessible support materials (Langegård et al. 2021; Mukasa et al. 2021; Singh et al. 2021), and interactive online learning community where students can exchange experiences and assist each other to navigate the platform to create confidence and friendships (Divjak et al. 2022; Langegård et al. 2021; Singh et al. 2021). By combining these strategies, the perception of online learning management systems among students can improve, resulting in a more enjoyable experience.

## Conclusion

This study sheds light on the perceptions of blended learning among nursing students and highlights the importance of preparing students for blended learning experiences focusing on the blended learning process, the importance of blended learning content alignment, and general usability. Targeted interventions are essential for optimising blended learning implementation in nursing modules highlighting considerations for educators and institutions navigating the growing environment of blended learning in nursing education, providing recommendations on how to maximise its benefits while addressing accompanying obstacles.

### Limitations of the study

The methodological approach was limited to a single university, and the participants were from a specific country. Additionally, convenience sampling was used, and the knowledge of the students was very specific.

### Recommendations

It is essential to provide comprehensive training and familiarisation of students with blended learning prior to embarking on offering a previous face-to-face module in a blended learning format. Additionally, investing in the professional development of nurse educators is crucial to ensure that they can develop effective online learning activities. Lastly, the importance of user-friendly and intuitive technological platforms is essential to enhance the overall student experiences.
